# Acceptance and Resistance of New Digital Technologies in Medicine: Qualitative Study

**DOI:** 10.2196/11072

**Published:** 2018-12-04

**Authors:** Sabur Safi, Thomas Thiessen, Kurt JG Schmailzl

**Affiliations:** 1 Medical School Brandenburg digilog Center for Connected Health Care UG Neuruppin Germany; 2 BSP Business School Berlin digilog Center for Connected Health Care UG Berlin Germany

**Keywords:** innovation diffusion, information and technology communication, telematics infrastructure, health care innovation

## Abstract

**Background:**

This study discusses the acceptance of new medical technologies in health care settings and resistance to these technologies from hospitals, doctors’ surgical centers, electronic health (eHealth) centers, and related institutions. We suggest a novel method of identifying factors that influence the acceptance of, and resistance to, new technologies by medical staff and patients.

**Objective:**

The objective of this study was to determine and evaluate the factors that influence acceptance and resistance to achieve a successful implementation of new technologies.

**Methods:**

The target group was patients residing in Brandenburg and major stakeholders in the local health care structure, for instance, medical institutions and medical professionals. The process relies on 3 models: the technology acceptance model, the unified technology acceptance and use of technology model, and the theory of technical innovation diffusion. Qualitative methodology was employed in this study, and an exploratory design was adopted to gain new insights into a poorly understood phenomenon in the German context. This enabled the researcher to take a flexible approach toward exploring a wide range of secondary data and to choose a different approach when unexpected information emerged. Content analysis was used to identify and interpret the data, and the researcher assured that the meaning associated with the information has concurred with that of the original source.

**Results:**

This study confirmed that adoption of new technologies in health care depended on individual opinions of the factors relating to them. Some medical professionals believed that technology would interfere with their ability to make independent diagnoses and their relationships with patients. Doctors also feared that technology was a means of management control. In contrast, other medical staff welcomed technology because it provided them with more opportunities to interact with patients and their carers. Generally, patients were more enthusiastic about technology than medical professionals and health care managers because it allowed them to have greater autonomy in selecting health care options. The need for all groups to be involved in the development of the new health care approach was an important outcome, otherwise resistance to it was likely to be greater. In other words, the strategy for change management was the indicator of success or failure. Therefore, following our analysis, a number of practical precepts emerged that could facilitate user acceptance of digital solutions and innovative medical technologies.

**Conclusions:**

The acceptance of digital solutions and innovative medical technology by patients and professionals relies on understanding their anxieties and feelings of insecurity. The process will take time because individuals accept change at different rates. Hence, the development of an extensive user community to fully and successfully implement eHealth is less likely in the short term; however, this should not prevent the push for changes in health care technology.

## Introduction

The term electronic health (*eHealth*) encompasses all apps that integrate modern information and communication technologies (ICT) to treat and care for patients. Therefore, eHealth is considered to be a general term for a wide range of ICT-based applications, which process information electronically. This information can be exchanged to support patient treatment and care processes because medical data obtained from the eHealth card can be easily communicated. This information consists of emergency data, treatment plans, medications, and the electronic patient file or telemedicine applications. This health information is communicated via a telematics infrastructure [1], and eHealth is summarized as follows: “a new term used to describe the combined use of electronic communication and information technology in the health sector” [2].

Therefore, eHealth centers are institutions or workstations that are fully equipped with specific medical diagnostic equipment and information technologies (ITs). These features enable members to run diagnostic tests and communicate results to doctors in real time. Thus, eHealth centers demonstrate how modern health care can be delivered to underserved populations and can encourage healthier communities.

Health care innovations seek to achieve stability, security, sustainability, and high qualitative value through networked structures, technological solutions, and analog interaction spaces. Innovative medical technology and digital solutions are components of innovative patterns and models. However, the successful implementation of innovative medical technologies, in particular digital companions, depends on acceptance by medical staff, such as doctors and nurses. These individuals directly confront new technologies and their implementation, with patients positioned as customers. To determine if innovation benefits these groups, resistance must be identified and reduced by creating greater awareness of the technologies and convincing potential users of advantages associated with the use of these technologies. Therefore, in medical settings, the implementation of new medical technologies should also consider psychological indicators that indicate developing acceptance, thus supporting a win-win situation. Resistance to new technologies or procedures should be recognized by medical professionals and their customers. Once recognized, these forms of resistance can be overcome by carefully planned and appropriate interventions.

Therefore, we sought to identify drivers of and barriers to the adoption of new health care technologies by referencing existing studies and then generated recommendations for improving technology uptake and diffusion. The resultant recommendations will be tested in future research projects.

Acceptance is a perceptual phenomenon that involves evaluation of new experiences and arriving at a final decision with respect to the benefits and limitations of that experience. Acceptance outcomes are derived from attitudes or courses of action. The development of acceptance depends on the interaction of 3 elements: subjective acceptance, objective acceptance, and the context in which acceptance occurs. Acceptance is an unpredictable construct because modified perceptions or general conditions can lead to different levels of acceptance. The decision to accept or reject a certain technology depends on various influencing factors [3]. Psychological approaches focus on attitudes, positions, norms, and value system factors that influence acceptance. Emotions and sociodemographic factors, such as age, gender, and educational level, similarly influence acceptance. Objective acceptance relies on how relevant an individual evaluates an innovation’s characteristics, acknowledging that identical characteristics may lead to different responses. This is because major influencing factors vary among individuals and may include financial impact, cost-benefit analysis, acquisition of necessary skills, or opportunities for work facilitation.

Technology acceptance also depends on perceived risks, such as whether the technology delivers secure care that is reliable and effective. Ease of use is of particular concern to users as new technology should facilitate more efficient execution of health-related tasks. The content factors related to acceptance are not directly related to users; rather, they externally influence users. One example of this could be jobs that are supported by new technologies and social processes in organizations, groups, or communities involved in implementing new technologies. Other contextual factors include organizational and social environments, including existing routines, political climates, participation cultures, the state of the economy, legal frameworks, and the processes by which innovation is introduced.

The technology acceptance model (TAM) is frequently used to explain acceptance [4] and provides insight into an individual’s decision to use or reject technological innovations [5]. The TAM proposes that the use of technology depends on 2 variables: how useful the technology is perceived to be and how easily it can be employed. Perceived usefulness is defined as an individual’s subjective evaluation of the new technology in relation to how much it will enhance their job performance. In contrast, perceived ease of use is the assessment of the effort required to learn and use technology. The balance of effort and usefulness underpins the development of user acceptance and influences user motivation. In general, an individual’s motivation to use a technology is higher if that technology is easy to use. External factors, such as support measures, have a positive effect on the perception of usefulness and on understanding a technology. In general, individuals adjust to new procedures quickly.

The unified technology acceptance and use of technology (UTAUT) model is an extension of the TAM that bases the growth of acceptance on 4 factors: performance expectancy, effort expectancy, social influence, and facilitating conditions [6]. Performance expectancy is a person’s perception of the extent to which a new technology brings about improvement and is the strongest predictor for the development of acceptance. As with the TAM, effort expectancy in the UTAUT model is the perceived usefulness and complexity of the technology. Social influence describes an individual’s perception of the extent to which others believe a new technology should be used and facilitating conditions under which individuals recognize interventions that support the use of the technology, for example, organizational or technical infrastructures. These models are used more than any other methods to explain acceptance, and some technology specialists suggest that studying their effectiveness has detracted from new research fields in technology acceptance. Their major shortcomings include the fact that research related to TAM was conducted under the assumption that a positive relationship exists between new technology use and user satisfaction, quality, and productivity; however, this was not proven [7].

The innovation diffusion theory examines technology uptake by individuals and organizations, focusing on the technological innovation development process from the invention stage to general acceptance or rejection. Five characteristics of innovation influence the diffusion of a new technology: relative advantage, compatibility, complexity, opportunity for a trial, and observability. Relative advantage is a technology’s perceived superiority over current methodologies, whereas compatibility is a social factor related to how well the technology matches social norms and behaviors. The remaining 3 factors describe its practical usage, with complexity indicating how easy it is to learn, opportunity for a trial being the technology’s amenability to evaluation and the chances of this occurring before adaptation is determined, and observability is associated with the capacity to observe the new technology’s outputs and advantages over alternatives. Although all 5 factors affect the rate of technology diffusion and no single aspect is strong enough to predict acceptance, the rate of innovation diffusion is the most affected by low complexity, the opportunity to *try out* novel technologies, and observability [8]. Empirical studies have also found that relative advantage and compatibility have positive associations with technology adoption, whereas complexity is negatively related to adoption [9]. The limitations of the innovation diffusion theory include the interpretation of relative advantage, which is a subjective factor because a cost versus benefits comparison is most important to some individuals, whereas ease of use is more appreciated by others.

The indicators of the development of acceptance, characteristics of the actors, features of the technological innovation, planned interventions implemented for its introduction, and observable benefits for patients and practitioners (including social factors and diverse environmental characteristics) decide the likelihood of individual adoption and dissemination of innovations. The weaknesses associated with the 3 theories, however, represent a gap in the current literature. There is a need to verify whether the assumptions underlying TAM result in positive outcomes and whether the subjective nature of relative advantage affects the rate of technology uptake purported by the innovation diffusion theory.

As environmental factors do not change easily, they are considered to be exogenous. Endogenous components, such as the actors and innovation, are critical for the development of acceptance. These factors are explored in the proposed methodology.

## Methods

### Methodological Process

This study is the precursor to a future project; therefore, the methodology indicates how the entire study will proceed. This study gathers major findings from previous research on eHealth technology acceptance to make recommendations for enhancing uptake of technology diffusion, which will be later tested empirically.

### Research Philosophy, Design, and Strategy

Social science research may adopt 2 diverse philosophies: objective and subjective stances. The objective stance seeks to determine objective cause-and-effect links between variables, whereas the subjective approach seeks to gather deep insight into how human beings interpret the same phenomenon differently. The objective approach is associated with a positivist research philosophy, which is similar to scientific approaches. It suggests that acceptable knowledge is generated from 1 source, that reality based on the phenomenon is independent of its context, and that the researcher has no effect on the outcomes of their research work; in other words, the research findings are value free. This stance is less important to resolving the research problem in this study, but its cause-and-effect links do form part of the solution. However, the subjective reasons as to why these links occur are of more practical value to health care providers and their patients; therefore, the emphasis of this study is on the subjective stance.

The subjective stance assumes that knowledge is derived from many sources because individuals observing the same phenomenon will tend to interpret it in diverse ways. These individualized interpretations exist as a consequence of each individual’s diverse values, beliefs, and experiences. Hence, reality is socially constructed, and the researcher is a major part of the study; thus, research is value-laden. Traditionally, although research has been based on either an objective or a subjective stance, researchers have recently focused on a combination of both stances; however, these combinations can vary in their relative proportions. Therefore, the main research design for this study is exploratory, which means that the researcher takes a flexible approach toward obtaining in-depth insights into a new or poorly understood phenomenon, as is the case in this study. If the researcher discovers unexpected information, this information can be pursued and the direction of research altered. In contrast, an objective stance demands stringent adherence to a pre-established design, which can be of much less value for answering certain research questions. The study, which has 2 parts, intends to generate new findings, an inductive approach, while confirming known theory, which is referred to as deduction [10].

The research philosophy is, therefore, interpretative as the subjective and objective stances can be combined in 1 study [11]. The research strategy for this study is archival and documentary because secondary data are used to establish the current status of technology acceptance in eHealth. However, moving forward, we will adopt a survey strategy using semistructured interviews to gain the opinions of medical staff and patients on the basis of the recommendations made by this initial study.

### Methodology for Data Collection and Analysis

Qualitative research methodology, which is well established with the interpretivist research philosophy and with the theory-building inductive approach, was selected for the study. Therefore, qualitative data will be the main priority; however, some numerical quantitative data may also emerge.

In this study, secondary data will be collected from robust sources such as books, journal articles, specialist magazines, and reliable websites that are noncommercial. These archived data are freely available in electronic form and comprise specialist articles, quality newspaper reports, and interview transcripts, for instance [10]. The future study will employ semistructured interviews for the collection of empirical data suitable for identifying resistance from employees and patients in Brandenburg, Germany. In this case, feelings of fear and insecurity can be identified by means of standardized interviews as part of the development of acceptance because interview questions will be based on the findings of this study.

### Sample

The sample for the second study is a purposive, nonprobability sample that uses the opinions of experts to answer the research question; the researcher selects the interviewees and invites them to participate. In-depth knowledge is more important than the generation of findings that are applicable to an entire population, particularly because populations in different communities are likely to hold diverse views [11]. The sample comprises a combination of the key stakeholders in the Brandenburg local health care structures and its patients, including doctors’ practices, accident and emergency hospital departments, medical professionals, and other health care professionals. Local health care structures play a key role in technology adoption because they can serve as multipliers and act as partners for digital care solutions. The communication target groups, which are the focus of efforts to build new technology acceptance, include key medical opinion leaders: The Brandenburg Chamber of Physicians; The Brandenburg Association of Statutory Health Insurance Physicians; Associations of General Practitioners in Brandenburg; other relevant professional and trade groups; and representatives of the local, county, and state governments.

Data analysis for this study and the empirical study to test its findings will be conducted using content analysis. This means that the data collected and transcribed will be scrutinized to identify keywords and phrases that are associated with technology acceptance and how the development of acceptance might be enhanced and its diffusion rate accelerated. These words and phrases will be organized into major themes, which are interpreted for meaning by the originator or originators, and can then be discussed, summarized, and presented in tables and charts [12].

### Ethics

All ethical standards associated with social science research are applied to this study. Although the first part is solely informed by secondary data, the researchers aim to interpret the data such that it reflects its original emphasis rather than the researchers’ own preferences. In the future empirical study, the researchers will ensure that participants do not suffer any harm as a consequence of expressing their opinions, and strict confidentiality will be maintained; in other words, the report will not indicate the source of any view expressed within study interviews [11].

## Results

The increased number of patients being treated is generally caused by the aging society, which incremented the complexity of health care systems. This is due to the number of terminal diseases experienced by these patients and the introduction of new technologies that enable medical professionals to diagnose illnesses more precisely. These technologies also give rise to surgical interventions that are more effective and less invasive. However, to benefit from new technology app, sustainable financial resources must be first organized in a cross-sectoral manner in primary care institutions, specialist clinics, and rehabilitation centers. However, the concept of a *boundary-less hospital*, although achievable, is hindered internally by insufficient, ineffective network design, silos resulting in poor communication, lack of an interdisciplinary approach, and inefficient processes. eHealth services have the potential to resolve the challenges of treating increasing numbers of patients, including those with chronic diseases, and creating efficient communication between departments. Many studies have demonstrated the benefits of telemonitoring, reducing hospital emissions, and controlling chronic conditions remotely for patients. However, despite these positive facts, uptake has been slow [13] because the potential cost-saving advantages of new technologies are not always evident to major stakeholders. In some cases, new technologies that comprise eHealth solutions are initially associated with higher costs and more time compared with traditional alternatives [14].

Major influencers in the adoption of eHealth are reported in empirical studies, for instance, the extent of trust that the patient has in a service provider, perceived user-friendliness of tools, health condition severity, and anonymity when using self-diagnosis tools. Medical professionals have concerns regarding the design of eHealth services and the technologies on which it will rely. Medical professionals also hold subjective opinions of the usefulness of new technology, its complexity, and/or how familiar technology is to end users. Hospital culture, location, and size have impacted the decision makers’ consideration for the relevance of tools such as eHealth applications for radiology and patient scheduling. Hence, there are 3 groups of main stakeholders comprising important subgroups, and these subgroups affect both acceptance and the development of acceptance.

Empirical research on patient acceptance factors affirms the importance of age. Although older people tend to need health care services the most, this group is often averse to technology, to the point where customized interventions are needed to support tool adoption. Despite widespread adoption of mobile technologies, such as smartphones, in Germany, with millions of people downloading apps, very few older adults use eHealth apps, preferring websites and email [15]. This suggests a lack of awareness of the benefits of these applications. In general, the study demonstrated that acceptance is a multistage process and that patients developed acceptance according to defined stages and at different speeds. Various organizations and medical professionals serve to raise awareness within the health care system; therefore, service providers should increase their marketing efforts. This might include highlighting benefits to patients through enhanced communication with medical professionals and greater access to support and 24/7 monitoring of known illnesses. Medical professionals could also leverage patient awareness of the potential for individualized service because they hold access to electronically organized patient information, which can be continuously updated.

Medical professionals and organizations could also inform patients of reliable medical websites, which provide information on the benefits and costs of eHealth. In addition, health service and medical professionals should elicit feedback from patients to support more effective use of eHealth tools and help improve the quality of these tools. In effect, patients need to be involved in the development of eHealth acceptance [13].

eHealth cards were introduced in Germany approximately 10 years ago upon their mandated use. An empirical study found that primary care doctors felt that eHealth could lead to fewer prescription errors and improve communication among various individuals and groups providing patient care. However, doctors also stated that their involvement in technology development and their ICT expertise were very low. The study also found that 46% of the variance in the perceived usefulness of eHealth cards was related to IT capability [16]. Health care professionals’ motivation to use eHealth records depended on the quality of interaction with the patient; however, lack of time, workload volume, perception of technology as a major threat to medical professional autonomy, and potential use of technology as a management control tool were significant barriers, according to a systematic literature review of 52 studies [17]. The extent of IT support and training had a substantial impact on the acceptance and implementation of eHealth technology by medical professionals. If there was no standard process and procedure for the health care organizations at the local, national, and regional levels, doctors and managers were less motivated to use the system.

Patients tended to be more positive about eHealth technologies than the other 2 user groups, recognizing that they had autonomy in their health management. If managers simply imposed eHealth techniques and processes for health professionals and other staff, the failure rate was high. In contrast, when a planning and implementation process involved user groups and a bottom-up development system, enthusiasm and commitment were generated. Hence, the actual change management process was the driver of success or failure. This review also identified that the most frequent reasons for acceptance of eHealth records were design, technical concerns, privacy and security, capacity for fully integrated health information systems within and across organizational boundaries, ease of use, costs, familiarity, and productivity. A total of 4 health care user groups were the subject of 3 linked studies: doctors, other health care professionals, health information professionals, and managers [17]. The participants were asked to rate the importance of and potential for implementation of 10 factors. Here, participants agreed that importance and applicability were criteria for success. There was a high agreement among managers that interoperability and outcome expectancy were the most important factors, whereas high levels of consensus among health care and health information professionals focused on perceived usefulness, productivity, motivation, and participation of end users in implementation. In addition, although health care professionals agreed that patient and health professional interaction, time constraints, workload, and available resources were important, an additional area of high agreement among them was management. These findings illustrate the differing priorities of the user groups, who therefore have different roles to play in the implementation process.

The volume of data associated with eHealth necessitates greater cognitive effort and creates a higher administrative burden. Consequently, many key players perceive eHealth solutions as an additional time-consuming effort rather than as a source of useful applications. Moreover, 1 reason for this opinion is that these individuals were not invited to participate in the process of developing technical solutions, and therefore, their needs were not considered. Consequently, they failed to understand how innovation supports their daily tasks. In Germany, awareness of eHealth solutions is lower than that in other countries. Therefore, new medical technologies are not widespread, leading to an information deficit. Older adults are not sufficiently familiar with telemedicine supplies and products, and this lack of awareness is aggravated by the paucity of cross-functional interactions among various health care sectors. As no reliable and protected nationwide infrastructure exists, deficiencies in the quality of care and efficiency of administrative and delivery processes occur. Manual collection and transmission of data also generate administrative delays and can be a source of error, which results in the real potential of eHealth solutions being underappreciated. In most cases, low technology–related expectations act as a self-fulfilling prophecy because major stakeholders, such as doctors, imagine that their peers will not fully support eHealth solutions and will not exchange data consistently. Therefore, developing an extensive user community to fully and successfully implement eHealth is less likely to occur in the short term [18].

## Discussion

### Principal Findings

The discussion of the research findings focuses on the challenges to the effective development of acceptance, which were revealed by this study and compared with the theoretical framework presented in the Introduction. In health care, the decision to use or to avoid new technologies depends on various factors, as suggested in previous studies [3]. Although some factors are common to patients, medical professionals, and health care organizations and their managers, there exist substantial differences in users’ perceptions of the importance of each factor. This observation agrees with earlier research that posits that decision making regarding acceptance is a subjective process [4,5]. For example, some doctors expressed concerns that technology could affect professional autonomy while diagnosing or treating patients. Another concern was that the organizations might use eHealth tools as means of controlling doctors. These perceptions succeed in generating negative attitudes toward implementing change. Doctors perceived technology as a positive factor by potentially reducing errors when prescribing patient treatments and as a means of improving communication with other groups and individuals caring for patients. However, doctors stated that their involvement in the development of technology and their ICT expertise were very low. The study also found that 46% of the variance in the perceived usefulness of the eHealth card related to IT capability [19].

Importantly, there were different levels of agreement among user groups on the 10 criteria considered to be important for eHealth adoption. Outcome expectancy and interoperability were the most important to managers, whereas perceived usefulness, productivity, and motivation were important to health care professionals. However, there was a high consensus among medical professionals regarding the importance of patient-carer interactions, available time, workload, and available resources. Interestingly, they also emphasized the importance of end-user involvement in implementation. Managers and medical professionals considered that the lack of standardization and integration among health care systems was a huge demotivating factor for eHealth implementation; therefore, this was not a facilitating condition. Based from the UTAUT model, the social condition factor is the most important aspect as it is represented by the age of patients. This factor therefore measures the complexity of the technology when compared with traditional methods shown by the way change was introduced, which can either be imposed by a top-down process or ushered in by a bottom-up process [6]. Ease of use was a general factor that was reinforced by these findings and support measures such as IT training and support for medical professionals, as reflected by the TAM [4,5]. Doctors also suggested that their involvement in technology implementation was low and that ICT expertise was an issue for them (and for all stakeholders generally) because the capacity to use the electronic system had a 46% effect on how useful they felt the technology to be. These findings also align with the innovation diffusion theory [9] in terms of relative advantage, perceived complexity, time, and opportunity to evaluate the technologies while undergoing training, with the opportunity to observe its potential advantages indicating how easy it is to learn. The opportunity to evaluate technology before deciding whether to adopt it and the observability of technology-related advantages were also inferred in the training and support that health professionals felt were necessary for acceptance. This study also suggested that the patients were encouraged by the eHealth-related capacity for health self-management; however, trust in the care provider, system’s ease of use, severity of the medical condition, and data security were additional concerns [6]. In addition, technology did not appear to be an issue for patients because they had already used it; rather, issues centered around lack of awareness of the usefulness of the technology.

### Recommendations for the Planned Electronic Health Center

The findings of this study have generated a range of recommendations for the planned eHealth center, which are presented in this final section.

The major stakeholders, who will be the users of the telemedicine processes, must be involved in the design of the eHealth centers and associated technologies. All those who are involved should be active participants in each phase of the innovation process as part of responsible research and innovation. To make an informed contribution, all medical professionals need to be informed about the major features of the innovation and its major benefits, especially effective treatment of more patients, with lower effort per patient. A transparent, accurate, user-centered ICT strategy that acknowledges feelings of insecurity and ensures that the information provided meets the needs of the target group must be devised. Merely instructing users on how to use the technology is insufficient for gaining their interest and commitment. Transfer of knowledge and skills in terms of the practical impact that technology can have on health care outcomes must be an integral part of the learning process. The implementation strategy must also include interventions that build a positive attitude toward the technology among various target groups of patients and the general population. Hence, the implementation strategy must be integrated into the overall eHealth strategy with a prolonged rollout period. This will enable all stakeholder groups to adapt and acknowledge the fact that technology diffusion occurs at different rates. Involving all stakeholder groups appropriately in the development of the change interventions will reduce their resistance to change and enable introspection of the groups’ perceived barriers to implementation. The advantages will become more observable to each group, and the realization that they each have different ideas about what those advantages are will be better appreciated. Individuals responsible for the implementation process must be regarded as trustworthy and proficient. This will encourage them to visibly demonstrate their support for the change and their role in accomplishing technology implementation.

With regard to the effective use of technology, professional learning and development personnel must introduce the various applications and explain their functionality to potential users, medical institution employees, and patients.

To address the perceived lack of cross-functionality, the communication among various key players must be improved and simplified. This could bring about a change in the traditional structures. The creation of a high level of acceptance through communication, participation, and support is an important condition for countrywide care delivery through eHealth solutions. Each innovation needs to be adapted to the wishes of the target group. Patient adherence is obtained after acceptance has been secured among employees of medical facilities, reinforcing the need for the acknowledgment of different rates of technology acceptance. Patients’ acceptance of innovation depends on their perceived ability to both use and directly benefit from it. Prejudices have a negative effect on patients’ readiness to deal intensively with *digital companions*, for instance. However, patient acceptance can be enhanced if doctors, surgery staff, community nurses, and other patients convey a positive perception toward the respective innovations. Conversations regarding change should take place with patients to increase their awareness and provide an opportunity to identify their resistance factors and overcome them. As patients’ positive perception depends on positive emotions and moods, their emotional participation must be encouraged, potentially through enjoyable elements that can be integrated into health care apps.

Patients’ acceptance of innovation could also be improved by offering sessions in health or community centers where the technology could be tested. Trained individuals would be available to offer support as the patients test the technical innovation. These settings also offer the potential for observability as other patients discuss the usefulness of the devices. During this time, a trained individual should be available to answer patients’ questions and explain the hardware and functions. A positive experience could be the first step toward patients developing a connection with the innovation because, in these settings, patients can be made to feel safe as their use is monitored. Users must interact with technologies and test their multipurpose options. Simulation environments can also be useful, including living labs. Technology users are important sources of information throughout all phases of product development. The simulation environment, with support from trained personnel, is important for developing acceptance, particularly among older adults residing in Brandenburg. Fortunately, these individuals are usually interested test users. The transdisciplinary experience data obtained from a living lab can subsequently be integrated into the structural concept of the eHealth center.

The demonstration of added value for patients and care providers is important, although some patients and medical staff react with skepticism to technical innovations and fear excessive external control. These feelings of insecurity among patients can be reduced by interactions with qualified and aware health care staff in living lab settings. Targeted health promotion through regional media, advertisements, or radio spots can create awareness of the advantages of eHealth centers. Related marketing objectives should include generating as much persuasion, memory value, and attention value as possible. Knowledge-imparting campaigns and information seminars that notify target stakeholder groups of relevant technology features are an additional option. As many patients view Web-based information before a consultation, there is danger that they could receive incorrect information or apply the information in the wrong context. The eHealth center gives patients quality-assured information and serves as an informational health platform; it can also recommend robust online sources.

As described in the Introduction, service providers view innovations in a positive way when the innovations have a positive effect on their daily activities. The acceptance of innovation among health professionals in Brandenburg will be achieved when the patient care goals are achieved more quickly and rendered at a higher quality through the use of new service delivery processes. This also applies to interface management and information flow. Employees will accept a technical innovation if resource use during service provision is lower and/or the revenue obtained by accessing new target groups is higher. Therefore, the eHealth center must help reduce professional burdens such as time pressure or the steadily rising mobility and documentation requirements in Brandenburg. Confident health professionals can convey their positive attitudes regarding innovation to their patients.

Conservative organizational forms, such as hospitals and their employees, often fail to easily adapt to technical innovations. Doctors, in particular, may hide their opposition to change. This could present a challenge for the planned eHealth center that is characterized by technological and procedural innovations. The benefits of innovation must be presented to hospitals in a very tactful manner; the reality of the need for economic efficiency exposes hospitals to challenges that relate to cost savings and competition. The eHealth center could help hospitals to stay competitive in the long run by facilitating the delivery of high-quality care while producing cost-effective services.

In view of the less-developed IT infrastructure in Brandenburg compared with other federal states, the creators of the eHealth center should lobby for internet access, broadband expansion, and rapid data transmission. Population groups with low digital affinity should be assisted in their efforts to acquire digital competence and suitable equipment through cooperation and coordination. Special attention should be given to data protection. Acceptance by the key players depends on the degree to which special encryption methods ensure the protection of personal data. However, patients with significant illnesses care less about the storage and protection of their data and more about their health care [20].

### Further Questions or Research Issues

The research on acceptance involves understanding the feelings of insecurity, fear, and apprehension experienced by different stakeholders. These feelings can be identified through suitable data collection processes. Comparing stakeholder perceptions is important to this ongoing study: initially, a sample from the target group will be interviewed about their feelings of insecurity and subjective perceptions of how technological innovations are implemented. Subsequently, the participants will be asked to test the innovation within a simulation or other environment that facilitates innovation testing. After a designated period, the participants will be interviewed again to reassess their feelings and perceptions. This process is intended to provide valuable insights into how testing an innovation positively influences key players’ perceptions.

### Conclusions

This research into the psychological indicators of acceptance shows that acceptance is critically dependent on the subject, object, and the general conditions that surround acceptance. In the case of the construction of an eHealth center, the acceptance subjects include target groups of patients (or the general population of individuals interested in preventative medicine issues) and medical professionals. The acceptance object is the technical innovation. The technical and social conditions are considered exogenous as they cannot be influenced easily or quickly. These general conditions are diverse. The first step toward securing the acceptance of digital solutions and innovative medical technology by patients and professionals is to understand their anxieties and feelings of insecurity on the basis of empirical study findings. This insight will create an opportunity to further categorize and evaluate the specific issues of the target group of disabled and elderly persons in the federal state of Brandenburg. The final step will be the generation of reliable recommendations for action for the eHealth center of the Federal State of Brandenburg. For both groups, acceptance can be generated only through a directed, transparent awareness campaign that provides users with sufficient information and the opportunity to test new technologies. Hence, users can directly experience the benefits of the technologies and acquire a positive attitude toward the new products.

## Abbreviations

eHealth: electronic health
ICT: information and communication technologies
IT: information technology
TAM: technology acceptance model
UTAUT: unified technology acceptance and use of technology model
